# FAIR and Interactive Data Graphics from a Scientific Knowledge Graph

**DOI:** 10.1038/s41597-022-01352-z

**Published:** 2022-05-27

**Authors:** Michael E. Deagen, Jamie P. McCusker, Tolulomo Fateye, Samuel Stouffer, L. Cate Brinson, Deborah L. McGuinness, Linda S. Schadler

**Affiliations:** 1 Department of Mechanical Engineering, University of Vermont, Burlington, VT USA; 2 Tetherless World Constellation, Rensselaer Polytechnic Institute, Troy, NY USA; 3 Department of Mechanical Engineering and Materials Science, Duke University, Durham, NC USA

**Keywords:** Materials science, Databases, Research management

## Abstract

Graph databases capture richly linked domain knowledge by integrating heterogeneous data and metadata into a unified representation. Here, we present the use of bespoke, interactive data graphics (bar charts, scatter plots, etc.) for visual exploration of a knowledge graph. By modeling a chart as a set of metadata that describes semantic context (SPARQL query) separately from visual context (Vega-Lite specification), we leverage the high-level, declarative nature of the SPARQL and Vega-Lite grammars to concisely specify web-based, interactive data graphics synchronized to a knowledge graph. Resources with dereferenceable URIs (uniform resource identifiers) can employ the hyperlink encoding channel or image marks in Vega-Lite to amplify the information content of a given data graphic, and published charts populate a browsable gallery of the database. We discuss design considerations that arise in relation to portability, persistence, and performance. Altogether, this pairing of SPARQL and Vega-Lite—demonstrated here in the domain of polymer nanocomposite materials science—offers an extensible approach to FAIR (findable, accessible, interoperable, reusable) scientific data visualization within a knowledge graph framework.

## Introduction

**Fig. 1:**
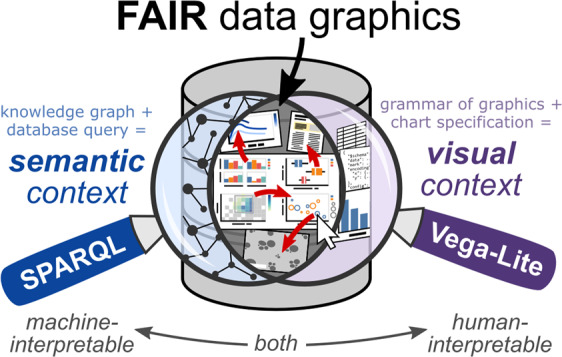
*Extending FAIR to data graphics.* In the paradigm of *charts as metadata*, a chart object is modeled as a set of metadata that includes semantic context (SPARQL query) and visual context (Vega-Lite chart specification). With the SPARQL query language and the Vega-Lite grammar of interactive graphics, one can specify interactive charts (bar charts, scatter plots, heat maps, etc.) that remain synchronized to the content of the knowledge graph and whose data marks can link to dereferenceable URIs (DOIs, images, other charts, etc.) through hyperlink encoding channels. Combined, these tools offer a human- and machine-interpretable way to explore and share scientific data.

From early cartography to modern digital interfaces, data visualization—the display of abstract information in graphical form—has helped humans navigate unknown and complex spaces with a history of conceptual advancements alongside innovations in printing and reproduction^1^. Today, the widespread availability of digitized information, and the ability to process and display it with computers and web browsers, has brought *interaction* to the fore as a facilitator of higher-level cognitive processing on multidimensional datasets^2^. Interactive data visualization supports human reasoning and understanding through iterative exploration and investigation^3^. Given the deluge of data in many scientific domains, human-interpretable means for managing, troubleshooting, and disseminating information—particularly those that preserve machine-interpretability—remain essential in scientific research. This article illustrates such an approach, on a knowledge graph database, through the combination of a robust visualization grammar (Vega-Lite) and the query language for the semantic web (SPARQL) (Fig. 1).

In response to challenges around the reuse of scholarly data^4^, scientific communities have mobilized around a set of four guiding principles for data management: *findable*, *accessible*, *interoperable*, and *reusable*^5^. Known by the acronym FAIR, these principles aim to preserve the value of digital assets through machine-interpretable metadata standards and schema. In the materials science domain, the FAIR guiding principles have been embraced by numerous data resources and repositories, ushering the development of modern data infrastructures for materials research^6–10^. The backbone and nervous system for these and other scientific data infrastructures build upon the foundation of the World Wide Web.

Since the early vision of the semantic web to make data on the Internet machine-interpretable^11^, the World Wide Web has evolved from a repository of linked documents to an omnipresent medium for information exchange. The resource description framework (RDF), a metadata model for the semantic web, captures knowledge through expressions known as *triples*, each comprising two nodes and a directional edge, that form a directed graph-based data representation inside a database, or triple store. SPARQL—a query language for RDF—uses graph-based expressions to retrieve sets of matches, or bindings, of variables in a graph pattern to content in a triple store. In the case of SELECT queries in SPARQL, sets of bindings take on a tabular form. The RDF model achieves interoperability through shared *ontologies*, or structured vocabularies that form the basis for capturing and reasoning over domain knowledge. Graph databases, such as *knowledge graphs*^12^, can build on the infrastructure of the internet by using uniform resource identifiers (URIs) that follow the well-established hypertext transfer protocol (HTTP) to ensure global uniqueness. Contrary to digital object identifiers (DOIs), which represent digital resources, URIs can represent anything (physical objects, abstract concepts, etc.). However, similar to the way a DOI is accessible via redirection when “https://dx.doi.org/” is placed in front, URIs can serve representations in a process known as *dereferencing*, offering a way to capture information stored elsewhere on the Web. Despite challenges around the implementation of truly distributed knowledge representations^13^, this extensible data and metadata format shows promise as a FAIR mechanism for storing and linking scientific data.

Several tools and platforms have been developed for exploring and visualizing RDF and linked data^14–21^, but a common thread in these systems is the use of a typology to define charts (e.g., bar charts, pie charts, scatter plots). Extensive research in data visualization has illuminated the deeper structure underlying most data graphics wherein graphical primitives known as data *marks* (e.g., point, line, area, text) have properties that can be encoded through *channels* (e.g., position, color, size, opacity) by mapping data *attributes* along discrete or continuous *scales*^22,23^. This *grammar of graphics* forms the basis for highly-cited and widely-adopted visualization libraries^24,25^. Reactive Vega^26^, and later Vega-Lite^27^, extended this grammar to interaction. In the Vega-Lite grammar for interactive graphics, a chart specification (written in JSON syntax) defines the visual representation of a tabular dataset (e.g., marks, encodings, selection parameters), while lower-level details (e.g., color schemes, legends, axis scales, event handlers) compile with default values unless overridden in the specification. The result is a concise, declarative specification of an interactive view of a dataset, built and customized incrementally.

Interactive methods for querying databases, such as Polaris and later VizQL (Tableau)^28,29^, offer platforms for authoring interactive charts and dashboards through drag-and-drop interfaces. These systems have provided significant value to business analytics with their ease of use and suitability for many common tasks, but they are restrictive in terms of their proprietary nature, limited expressivity, and lack of support for graph-based data sources. To counter these drawbacks and provide a means for FAIR scientific data visualization, we focus our efforts on use of available open-source tools, a high degree of expressivity, and compatibility with knowledge graphs.

In this article, we describe a paradigm wherein charts defined through metadata provide a mechanism for exploring and documenting the contents of a knowledge graph of materials science data. Building on the concept of a visualization as a function of a data storage medium and a user specification^30^, we model a chart as a combination of *query* (SPARQL) and *chart specification* (Vega-Lite) stored in the knowledge graph and processed on demand. This approach for bespoke, interactive data graphics is made possible by the high-level, declarative nature of SPARQL and Vega-Lite. Storing charts as metadata enables them to display the most up-to-date information in the knowledge graph, and charts themselves can be queried and analyzed. We find that *dereferenceable* URIs—HTTP identifiers that serve human-readable representations when opened in a web browser—embody the complementarity of SPARQL and Vega-Lite. Examples presented here draw from a knowledge graph in the materials science domain, but the paradigm applies to other domains as a mechanism for FAIR scientific data visualization and interaction.

## Results

By exploring the notion of charts as metadata, we find that the variety of bespoke data graphics offers a useful, interoperable platform for exploratory visualization of a knowledge graph.

### Sandbox for exploratory visualization, infographics, and meta-analyses

To address the trade-off between usability and expressivity, we opt for maximal expressivity in terms of content creation, taking usability into account by making all examples open-source and readily available for re-use. For example, domain experts without fluency in query or visualization languages (e.g., SPARQL, Vega-Lite) can interact with data in the knowledge graph by browsing a gallery of interactive charts, and those interested in creating their own charts have the code behind each chart as a precursor to adapt or modify for their own purposes. In this way, the collection of example queries and chart specifications provides a form of reusable documentation for accessing and viewing data in the knowledge graph.

To demonstrate the concept of charts as metadata, we extended the visualization capabilities of MaterialsMine (materialsmine.org) to accommodate the saving and processing of these bespoke data graphics. The knowledge graph at MaterialsMine, previously NanoMine^8,31^, contains curated data from research articles on polymer-matrix nanocomposite materials in the scholarly literature along with metadata describing the materials, processing, characterization, and bibliographic information from those articles. Structured as linked data conforming to semantic web ontologies and vocabularies^32^, data and metadata are made accessible through a SPARQL endpoint on the web.

Tailored interactive charts containing data from the knowledge graph range in purpose and complexity. Depending on the SPARQL query, datasets vary from individual sample data linked to a research article to meta-analyses of all articles curated into the knowledge graph (Fig. 1). All examples shown here use some combination of layered and concatenated views combined with selections in Vega-Lite to provide explorable, interactive views of data. Following the mantra of *overview first, zoom and filter, then details-on-demand*^33^, these data graphics use elements of interactivity to display aspects of a dataset that exceed the capability of a static representation. Common modes of interaction include tooltips, conditional display on hover interactions or selections, cross-filtered views, and pan and zoom.

**Fig. 2:**
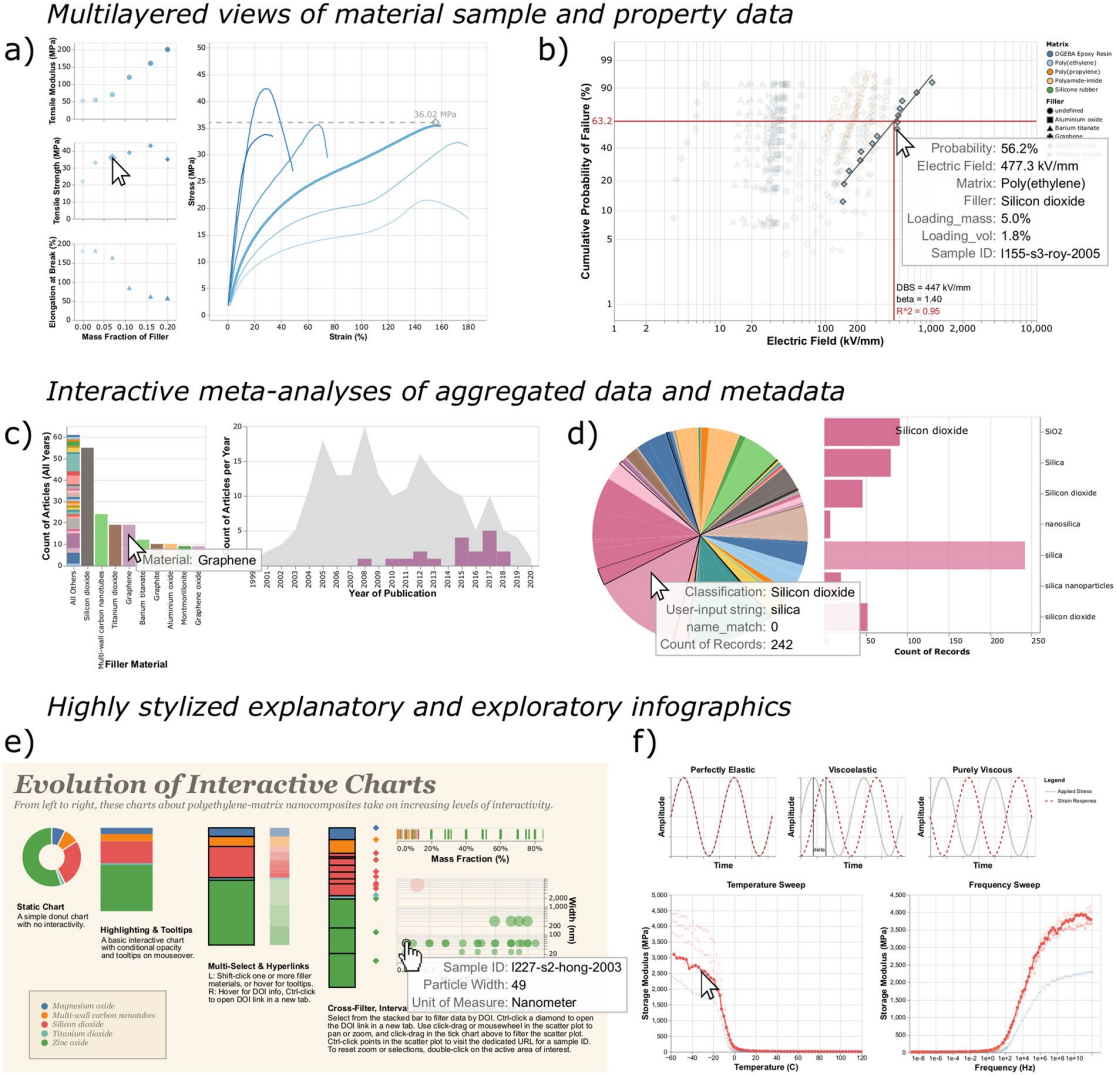
*Interactive views of sample data, meta-analyses, and stylized infographics.* Charts shown here are specified by a SPARQL query (semantic context) as well as Vega-Lite specification (visual context). The snapshots of interactive data graphics shown here display (**a**) mechanical tensile testing data curated from Bandyopadhyay *et al*. (2005)^48^, transformed into a layered composite view; (**b**) a Weibull plot of dielectric testing data using custom y-axis scaling and the regression transform to estimate dielectric breakdown strength (DBS); (**c**) a meta-analysis of nanocomposite filler materials in curated research articles per year of publication, highlighted to show the trend for graphene; (**d**) a meta-analysis of entity-resolved compound names (computed by the ChemProps API^34^) versus curator-provided strings; (**e**) an infographic showing a dataset with increasingly interactive views; and (**f**) an explanatory graphic for viscoelastic data. These examples created for the materials science domain represent a small subset of the variety of datasets and visualizations made possible by using SPARQL queries and Vega-Lite specifications to capture interactive views of content from a knowledge graph database.

Offering the full expressivity of SPARQL and Vega-Lite for specifying charts resulted in a number of interesting and often unanticipated interactive views of data in the knowledge graph. For example, rule marks with conditional opacity enable the overlaying of derived mechanical properties (tensile modulus, tensile strength, elongation at break) over representative curves showing raw tensile test data (Fig. 2a). Using Vega-Lite transforms and layered rule marks permits the custom scaling and plotting of linearized Weibull distributions for real-time calculation of dielectric breakdown strength (Fig. 2b). A query of articles and the material systems studied within them offers an interactive view of trends in polymer nanocomposite materials research (Fig. 2c). Another meta-analysis demonstrates the results of entity resolution with the ChemProps API (Fig. 2d)^34^. Concatenated sub-views and text formatting parameters result in a stylized infographic demonstrating some of the ways to enhance data exploration by adding interactive elements (Fig. 2e). In addition to concatenated sub-views, sequence generators and Vega-Lite transforms make possible an embedded explanation of dynamic mechanical analysis for viscoelastic material properties atop experimental data (Fig. 2f). These and over 150 other examples currently populate the gallery of charts in the MaterialsMine knowledge graph.

The examples presented here by no means represent the only way to query and display these data. By making available the expressivity offered by SPARQL and Vega-Lite, we encourage experimentation and rich customization in the pursuit of effective means of data exploration for a variety of applications. Any individual data visualization will have finite applicability. However, the collection of such open-source visualizations enabled by this approach can accomplish a variety of tasks and illuminate remote corners of a knowledge graph.

### Leveraging dereferenceable URIs in a knowledge graph

To avoid naming collisions, knowledge graphs employ URIs to globally identify resources without ambiguity. Using well-established internet protocols (e.g., HTTP) helps to ensure global uniqueness among distributed systems on the semantic web. A helpful practice for documenting resources involves the owner of a domain having a representation delivered by a server (e.g., HTML page) when a URI is requested through internet protocols. URIs can exist solely as identifiers, but those with available representations on the web are known as *dereferenceable* URIs.

**Fig. 3:**
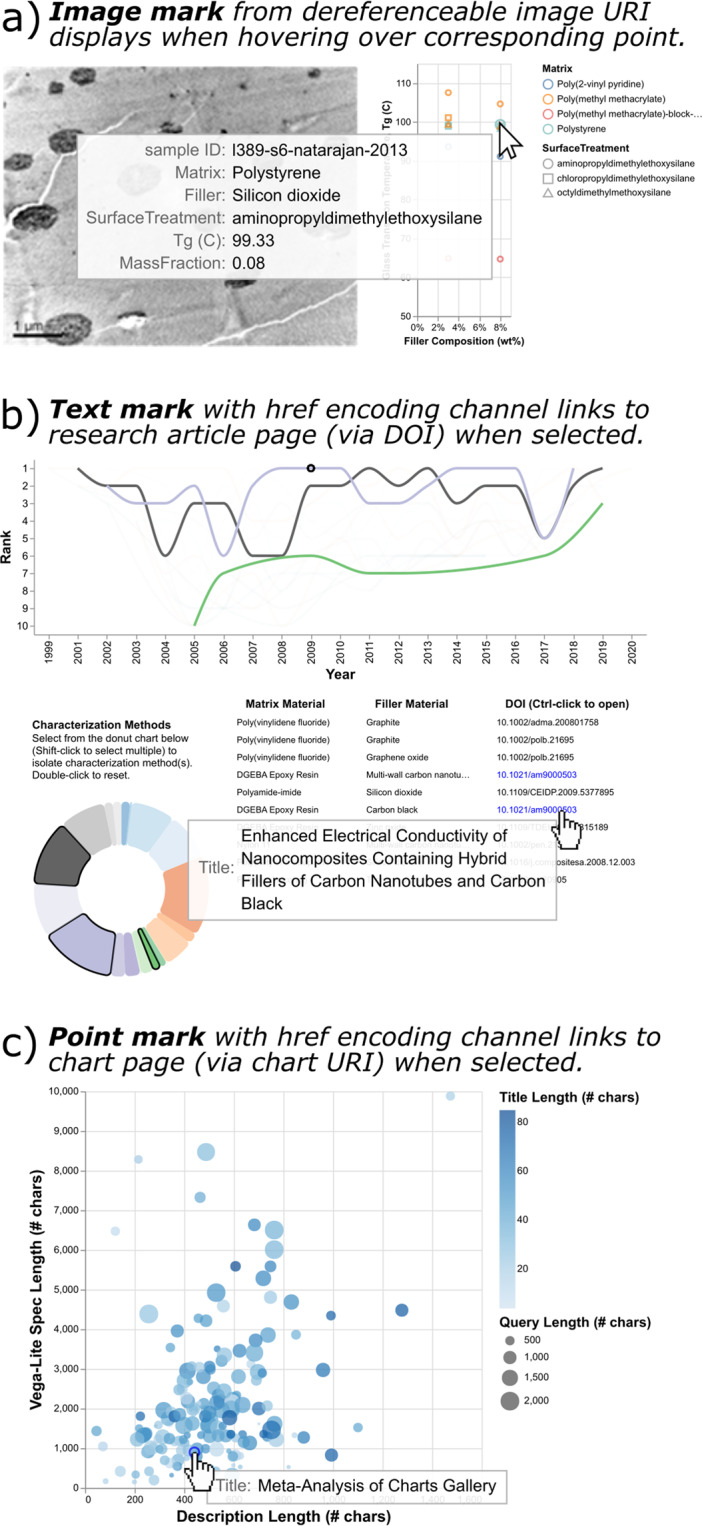
*Direct linking to representations of resources in the knowledge graph.* These charts make use of dereferenceable URIs in the knowledge graph to display or link to resources. (**a**) Image marks with accompanying URL encoding channels are used to display curated sample images from Natarajan *et al*. (2013)^49^ corresponding to the selected points on the adjacent scatter plot. (**b**) Text marks with a hyperlink encoding channel link open the URL of a journal article DOI when selected. (**c**) A scatter plot displays charts published to the knowledge graph, arranged by the character length of their Vega-Lite specification and description. Point marks with the hyperlink encoding channel link to a chart page when selected. This final chart is self-referential; the highlighted point mark represents the chart itself.

URIs can be returned in the results of a SPARQL query, but a column of URIs in a table may be less useful than an interactive visualization that allows a user to sort and refine the results of interest. *Overview first, zoom and filter, then details-on-demand*^33^. We identify two encoding channels in Vega-Lite that make the language well-suited to knowledge graphs: the *url* encoding channel for image marks (Fig. 3a), and the *href* (hyperlink reference) encoding channel for other data marks such as text (Fig. 3b) or point marks (Fig. 3c). First, images serve as useful visual representations in many scientific domains, and rendering them on-demand via dereferenceable URIs avoids the need to download or cache a full set of images. Second, the practice of hyperlinking to primary sources or representations leverages the notion of linked data by directing to additional information about resources outside the confines of a given chart.

Interactive data visualization offers myriad ways to explore a dataset, and we describe how knowledge graphs with dereferenceable URIs can expand the reach of these graphics to the entire Web through hyperlinks. By combining the strengths of knowledge graphs for storing knowledge and interactive visualizations for accessing knowledge, this approach provides a means for communicating data in a way that builds trust and makes data analysis more transparent, building on the idea that *sharing the graphic* should equate to *sharing the data*^35^.

### Interoperability with other web platforms

The semantic web facilitates data exchange in a distributed manner by building on the infrastructure of the internet and encouraging the use of common vocabularies and ontologies. One demonstration of interoperability enabled by SPARQL is the extension for federated querying. Federated queries aggregate data from multiple sources by running sub-queries across distributed SPARQL endpoints on the internet. Furthermore, the ability to send a query to a public SPARQL endpoint via HTTP GET request and receive machine-readable results (e.g., JSON) enables other web platforms to query and process data from a knowledge graph.

**Fig. 4:**
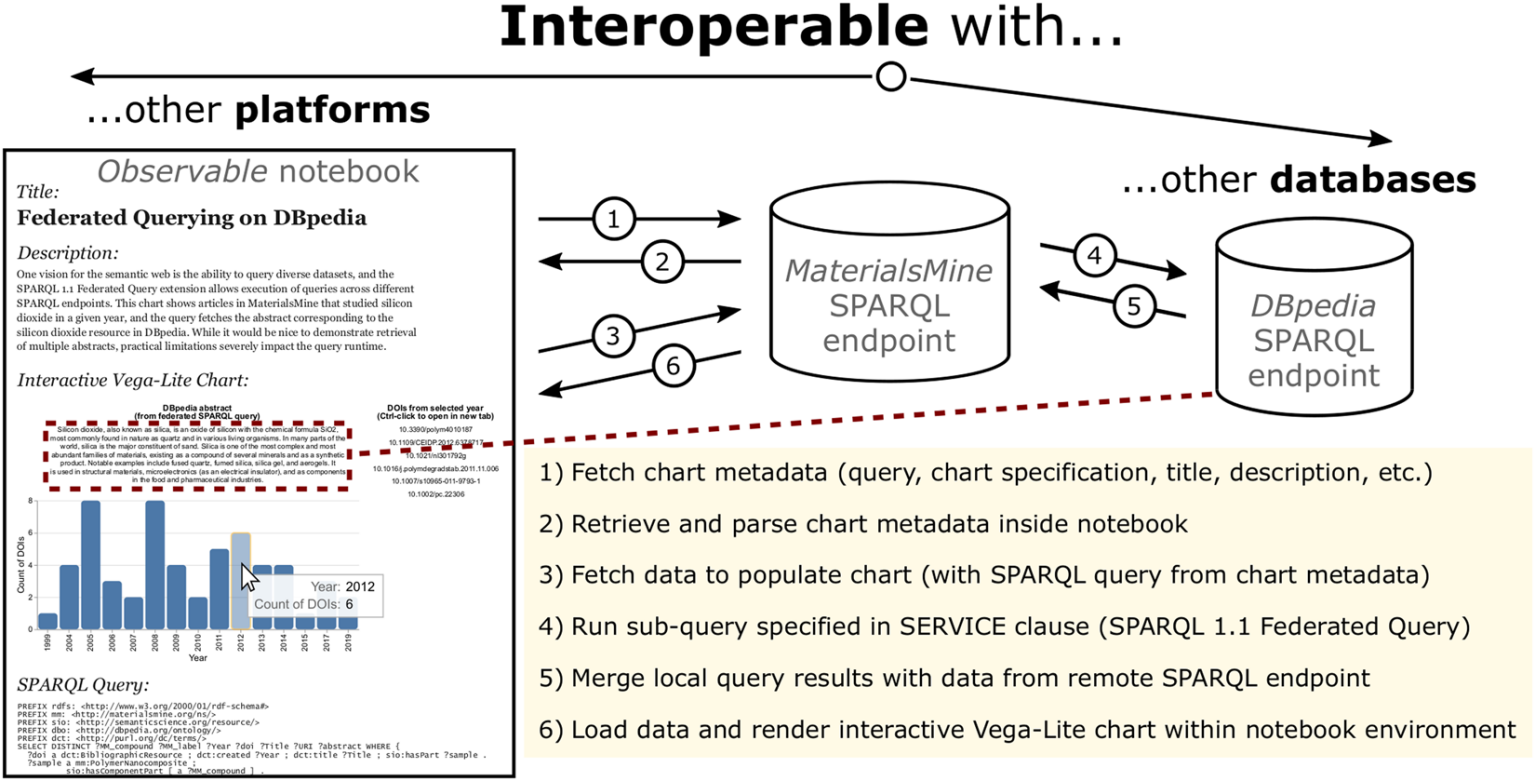
*Interoperability with other web platforms and FAIR data sources.* The ability of public SPARQL endpoints to send queries and receive data through internet protocols enables interoperability within a query (e.g., federated querying from DBpedia^36^) as well as displaying and processing information from the knowledge graph using external web-based platforms, such as an Observable notebook (https://observablehq.com/@mdeagen/figure-4-notebook).

Here, we demonstrate a two-fold example of interoperability by showing an example chart from MaterialsMine, with federated querying of DBpedia^36^, all within a reactive computational notebook on Observable (Fig. 4). Platforms such as Observable (https://observablehq.com), which natively supports Vega-Lite, can fetch a chart’s metadata, parse the query and chart specification, run the query for the chart’s data (in this case, at the same endpoint), then render those data as an interactive Vega-Lite chart. In this example, the query contains a SERVICE clause to the DBpedia SPARQL endpoint to return the English-text abstract for the material compound “Silicon dioxide” from Wikipedia, and the Vega-Lite specification displays this abstract as a text mark on the chart (Fig. 4, red dotted lines). At present, federated querying adds several seconds to the query runtime, therefore the development of such queries requires optimization.

Interoperability is arguably the most challenging of the FAIR principles to implement, and we have shown how a SPARQL-equipped knowledge graph can interoperate with other public SPARQL endpoints as well as display charts and their metadata on an external platform that supports Vega-Lite. In the Discussion section, we present design considerations for queries and chart specifications that arise in this approach to FAIR scientific data visualization.

### Decoupling (meta)data from graphical representation

Data graphics assemble and contextualize information for scientists, similar to how metadata package and describe data for machines. By choosing to model a data graphic (e.g., interactive Vega-Lite chart) as a form of metadata itself, researchers can simultaneously capture human-interpretable and machine-interpretable representations of their research output. This FAIR approach to data visualization leverages Vega-Lite’s grammar of interactive graphics, which differs fundamentally from conventional tools (Excel, Plotly, Matlab, etc.). By describing an interactive representation of data as a JSON object, a Vega-Lite specification illuminates the inherent structure of most data graphics, as opposed to a chart typology that requires many preset chart types to achieve expressivity. Upon introducing the ability to encode URIs as hyperlinks in data marks, Vega-Lite becomes an ideal tool for combining with semantic web technologies. While a formal grammar of graphics ontology falls outside the scope of the present work, such an effort could build upon these demonstrations of the reciprocal benefits of SPARQL and Vega-Lite and include stakeholders from both the semantic web and data visualization communities.

To further illustrate the benefits of the combined approach of SPARQL and Vega-Lite, we can consider the substitution of either tool with traditional alternatives. In the case of SPARQL with a typology-based plotting tool, one loses expressivity in terms of building interactive data graphics and may obscure the visual meaning captured in the rendered graphic. The inverse case—an isolated tabular dataset with a Vega-Lite specification—may lack sufficient metadata and semantic context necessary to interpret the raw data. With the combined approach, data and visual representations exist as metadata, with the added benefit that interactive charts can use hyperlink encoding channels to provide direct access to dereferenceable resources in the knowledge graph. Jointly, these tools embody FAIR scientific data visualization, and we elaborate further on the framing of specific FAIR guiding principles around these notions in the Methods section.

## Discussion

In the paradigm of *charts as metadata*, the data instances that populate a chart are absent from the chart specification. This may seem counter-intuitive, but the resulting specification describes *what* data to retrieve (i.e., semantic context) and *how* to display it (i.e., visual context). As a result, these metadata-defined charts represent *interactive lenses*, each with a particular vantage view of the knowledge graph, that display the most up-to-date instances from the knowledge graph at the time of rendering. Many approaches to designing static visualizations no longer apply when visualizations become interactive and subject to changing data^37^. We organize these design considerations into three broad categories: *portability*, *persistence*, and *performance*.

*Portability* poses a key challenge for web-based charts and interactive charts in general. The wide adoption of a given approach or toolset hinges on its reliability and compatibility with a diverse set of platforms and devices. To serve the intended use as a means for analyzing or disseminating data, an interactive data graphic must retain its ability to respond to user input when embedded in some other format (e.g., offline document, presentation slide), or offer a pre-recorded animation displaying its contents. Two recent projects, Chameleon and Loom, have begun to tackle some of these challenges around portability of interactive data graphics^38,39^.

*Persistence*, or the ability to continue existing as a useful data graphic, largely depends on the stability of the underlying data representations and their ability to be interpreted in the future. In a Vega-Lite chart specification, one may specify the schema used as a form of version control against future software changes in Vega-Lite. On the data query side, if vocabulary URIs or the way data are modeled in the knowledge graph change, SPARQL queries may cease to function as originally intended. We experienced this challenge when converting terms in the MaterialsMine ontology from their prior namespace (http://nanomine.org/ns/) to a new namespace (http://materialsmine.org/ns/). Updates to charts typically only involved a one-line change in the SPARQL PREFIX header of the query, but the issue highlighted the effect of upstream changes involving URIs on downstream resources such as charts. To mitigate these issues, communities should invest requisite resources to ensure robust ontologies and stable SPARQL endpoints that provide reliable access to data and a consistent semantic representation.

*Performance* of these charts may involve technological or data limitations. Query runtimes and chart rendering are necessarily impacted by the quantity of data available in the knowledge graph and how much data can be stored in memory. Moreover, responsiveness of public SPARQL endpoints, particularly with respect to federated querying, remains an ongoing challenge. For visualization and interaction design, accounting for future data involves considering how new data may impact scale extents, latency, or occlusion of data marks. Consideration of the scope of the data graphic becomes important, for example separating a large dataset into separate views that show a high-level view of the dataset with access to instances through interaction. *Overview first, zoom and filter, then details-on-demand*^33^. On a more technical note, rendering images in Vega-Lite requires the use of image URIs within the same domain or ensuring that images from external domains have the appropriate HTTP header enabling cross-origin resource sharing (CORS). Finally, scalability of a gallery of charts from a knowledge graph involves considerations of the ease with which domain experts can search and navigate the collection of charts available.

Relational and non-relational databases (e.g., SQL, NoSQL) provide limited account of the relationships between individual data objects, simplifying initial development of limited-scope data resources but hindering the later integration and interoperability with other data resources as these models and applications scale in complexity. Knowledge graph databases, on the other hand, use a graph data model upfront to capture these abstract relationships and semantics. Backend database performance still remains a concern when metadata employ a graph data model, but the use of shared ontologies mitigates the scaling issues around interoperability. When graph databases build upon the infrastructure of the World Wide Web and employ globally unique and dereferenceable identifiers (URIs), they lower the barriers for distributed data exchange and can benefit from a web-based interactive visualization grammar such as Vega-Lite.

Defining charts as metadata in a knowledge graph captures semantic context and visual context while providing interactive, human-interpretable documentation of the contents of a knowledge graph. These chart representations may also be considered a form of “visualization data,” an emerging data format relevant to the application of artificial intelligence (AI) to visualization generation, enhancement, and analysis^40^. The complementarity of SPARQL and Vega-Lite make this approach to scientific data visualization well-aligned with the FAIR principles by preserving machine-interpretability of underlying data while simultaneously providing an interactive means for domain experts to explore the contents of a knowledge graph.

## Methods

In this section we describe the metadata model for charts, show a minimal example of a functional chart, and describe how charts are created and managed.

### Metadata for a chart

**Fig. 5:**
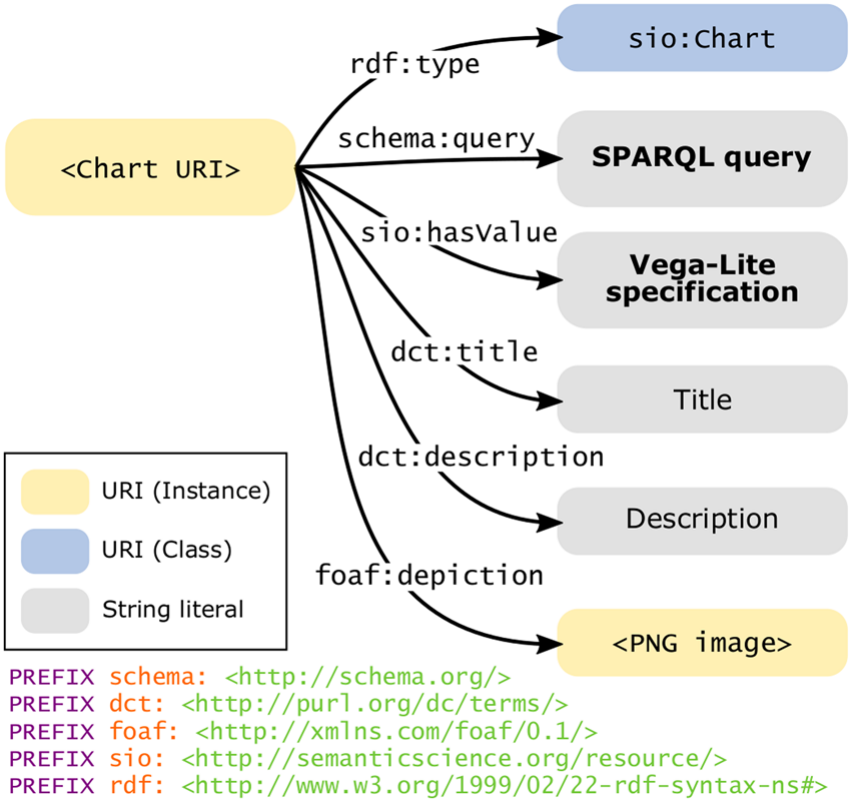
*Metadata describing a chart resource in the knowledge graph.* Each chart instance is a member of the class *sio:Chart*, with metadata including a thumbnail depiction (created at the time of chart publication) as well as string literals defining the title, description, query, and chart specification. URI namespace prefixes are shown at the bottom.

Expressing data queries and chart specifications as text allows them to be stored as string literals in the knowledge graph. We assign each chart URI to the class *sio:Chart* from the Semanticscience Integrated Ontology^41^, along with metadata corresponding to the widely-adopted Dublin Core, Schema.org, and FOAF vocabularies. In addition to the SPARQL query (i.e., semantic context) and Vega-Lite specification (i.e., visual context), we include a title, description, and thumbnail depiction of each chart (Fig. 5). When published to the knowledge graph, provenance metadata (when a chart was created and by which logged-in user) are captured as extensions of a named graph using the nanopublication framework^42^.

### Concise specification of chart metadata

**Fig. 6:**
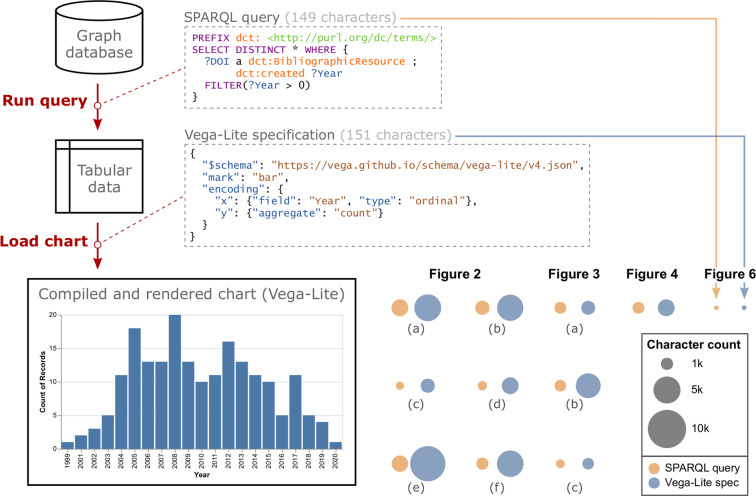
*Minimal example of a chart specification.* The bar chart in the lower left, showing the counts of curated research articles grouped by year of publication, was generated from a query and chart specification each containing approximately 150 characters. The faceted plot in the lower right, also generated using SPARQL and Vega-Lite, encodes character counts as the size of point marks to compare the relative brevity of this minimal example query and chart specification to other interactive, layered, and stylized charts featured in this article.

The raw data within a chart are not explicitly enumerated in its metadata but are instead captured implicitly via the SPARQL query. This method allows charts to accommodate data instances added to or updated within the knowledge graph at a future point in time. Here, we demonstrate a minimal (non-interactive) Vega-Lite chart that displays the count of research articles curated into the knowledge graph as a function of the year each article was published (Fig. 6). Combined, the query and chart specification require only 300 characters. Behind the scenes, the SPARQL engine processes the query to collect all available matches in the knowledge graph. The result of this query is a set of tabular data with two variable attributes (*DOI, Year*) which occupy nearly 10,000 characters if serialized as a single string. Tabular query results are passed to the Vega-Lite renderer, which processes the chart specification, performs an aggregation operation, formats the axes, and draws the data marks according to the specified encodings and default parameter values. As content is added to the knowledge graph, the tabular data returned by running the query will capture those new instances, and the Vega-Lite chart will reflect those data when compiled and rendered. Although the bar chart represents a minimal example, the figure shows how the sizes of the SPARQL query and Vega-Lite specification compare to other, more elaborate data graphics presented in this article.

### Browsing and creating charts

We use Whyis^43^, a Python Flask application for knowledge graphs, to upload and manage charts in the knowledge graph. All instances of *sio:Chart* currently populate a paginated gallery featuring the thumbnail depiction, chart title, preview of the description, and link to the chart URI. By clicking on a chart, a user is directed to a chart instance view which queries the knowledge graph, displays the chart title and description, and renders the Vega-Lite chart. Icons above the chart allow the user to view the SPARQL query and Vega-Lite chart specification. Given the many possible ways to visualize a tabular dataset, we also enable the user to explore the raw data returned by the query inside an instance of Data Voyager^44,45^, which provides a drag-and-drop interface for defining chart encodings and exploring recommended views.

To add a chart to the knowledge graph, a user enters the SPARQL query, Vega-Lite specification, title, and description into the custom chart editor interface in Whyis. SPARQL query syntax highlighting is enabled by embedding a YASGUI interface^46^. On the opposite panel of this interface, the user can toggle between views of the raw data as a table or Vega-Lite chart. When a chart is saved, a nanopublication is published to the knowledge graph, a backup of the chart metadata is created in MongoDB, and the chart joins the gallery of charts. At present, all charts published to the knowledge graph are publicly available. Future deployments may consider whether to offer tiered access or intermediate publication, for example saving progress on a chart under development or publishing with a limited scope (e.g., to a research team). In the meantime, development of charts can occur by using an offline platform such as Visual Studio Code with Vega-Lite plug-in, or by using an online platform such as Observable.

### Framing the FAIR guiding principles for data graphics

Here, we highlight several of the guiding principles of FAIR laid out by Wilkinson *et al*.^5^ and relate them to design decisions around the combined approach of SPARQL and Vega-Lite for scientific data visualization:

- *“F1. (Meta)data are assigned a globally unique and persistent identifier”*
  Chart objects, modeled as the combination of a SPARQL query and Vega-Lite specification among other metadata, are assigned a globally unique URI.
- *“A1. (Meta)data are retrievable by their identifier using a standardized communications protocol”*
  A chart object is dereferenceable through its HTTP URI, and data objects within a chart that have their own dereferenceable URIs can use image marks or the hyperlink encoding channel in the Vega-Lite specification.
- *“A1.1. The protocol is open, free, and universally implementable”*
  A public SPARQL endpoint provides access to the data, and the free, open-source software developed by the Vega-Lite community enables the rendering of valid chart specifications.
- *“A1.2. The protocol allows for an authentication and authorization procedure, where necessary”*
  To ensure provenance of chart objects, posting to the knowledge graph is limited to authenticated users who are logged into the web application.
- *“I1. (Meta)data use a formal, accessible, shared, and broadly applicable language for knowledge representation”*
  The RDF (meta)data model has been employed to capture the semantic relationships between a chart object, its associated metadata, and its provenance.
- *“I2. (Meta)data use vocabularies that follow FAIR principles”*
  We use the Semanticscience Integrated Ontology^41^ along with Dublin Core, Schema.org, and FOAF vocabularies.
- *“R1.2. (Meta)data are associated with detailed provenance”*
  The Whyis knowledge graph framework^43^, using the concept of nanopublications^42^, captures provenance metadata for charts posted to the knowledge graph, such as the creator and time of publication.
- *“R1.3. (Meta)data meet domain-relevant community standards”*
  Vega-Lite provides a high degree of expressivity, of which the figures in this article provide a small sample. The approach could also apply to scientific domains that utilize geospatial visualizations using Vega-Lite’s geographic projection abilities. Scientific visualizations outside of the scope of Vega-Lite (e.g., 3D models, molecular structures, annotated schematics, force-directed graphs, animations) can instead use static depictions with image marks and a hyperlink encoding channel to link to a more advanced representation.

## Data Availability

The “living” versions of these interactive charts, which require an operational SPARQL endpoint and Whyis application, can be found in the MaterialsMine Gallery of Interactive Charts at https://materialsmine.org/wi/gallery. As a backup, archival versions of each interactive chart featured in this article (using a static snapshot of queried data) are available on Observable (https://observablehq.com/@mdeagen/archival-interactive-charts). A zipped folder with the query and chart specification for each chart featured in this article, as well as a snapshot of the data retrieved by the query, is available on Figshare (10.6084/m9.figshare.19352258)^47^.

## Code availability

Source code for the Whyis application framework can be found on Github at https://github.com/tetherless-world/whyis/. Source code and documentation for the Vega-Lite project can be found on Github at https://github.com/vega/vega-lite. The W3C Recommendation for the SPARQL 1.1 Query Language can be found at https://www.w3.org/TR/sparql11-query/.
